# Increased lateral femoral condyle ratio measured by MRI is associated with higher risk of noncontact anterior cruciate ligament injury

**DOI:** 10.1186/s12891-022-05134-x

**Published:** 2022-03-01

**Authors:** Miao He, Jie Li

**Affiliations:** Department of Orthopaedic Surgery, Chongqing Emergency Medical Center (Chongqing University Central Hospital), No. 1 Jiankang Road, Chongqing, 400010 China

**Keywords:** Anterior cruciate ligament, Knee, Lateral femoral condyle ratio, Femur

## Abstract

**Background:**

Studies have shown a significant association between the radiographically measured lateral femoral condyle ratio (LFCR) and anterior cruciate ligament (ACL) injury. However, it is unclear whether LFCR measured by magnetic resonance imaging (MRI) is associated with a higher risk of noncontact ACL injury.

**Objective:**

To investigate the effect of LFCR on the risk of noncontact ACL injury by MRI. 2 to investigate the association of LFCR measured by MRI with multiple bone morphological risk factors and evaluate the most sensitive risk predictors of noncontact ACL injury.

**Methods:**

A total of 116 patients, including 58 subjects with noncontact ACL injury and 58 age-matched and sex-matched controls with only meniscus injury, were included in this retrospective case-control study. LFCR, lateral tibial slope (LTS), lateral tibial height (LTH), medial tibial slope (MTS), and medial tibial depth (MTD) were measured on MRI. The differences in each index between the two groups were compared, and risk factors were screened by single-factor logistic regression analysis. Indicators with *P* values < 0.1 were included in the logistic regression equation. The critical values and areas under the curve (AUCs) of independent risk factors were determined by receiver operating characteristic (ROC) curve analysis. Finally, the diagnostic performance of each risk factor was evaluated by the Z-test.

**Results:**

A total of 116 patients who met the inclusion criteria were included in the final analysis (58 cases in the noncontact ACL injury group and 58 cases in the control group). Patients with noncontact ACL injury had a higher femoral LFCR (0.64 ± 0.03) than patients with isolated meniscus tears. Among all the risk factors for ACL injury, the AUC for LFCR was the largest, at 0.81 (95% CI, 0.73-0.88), and when the critical value was 0.61, the sensitivity and specificity for the diagnosis of ACL injury were 0.79 and 0.67, respectively. When combined with LTH (> 2.35 mm), the diagnostic performance was improved. The AUC was 0.85 (95% CI, 0.78-0.92), the sensitivity was 0.83, and the specificity was 0.76.

**Conclusion:**

This study shows that an increased LFCR is related to an increased risk of noncontact ACL injury as determined by MRI. LFCR and LTH are sensitive risk factors for noncontact ACL injury and may help clinicians identify individuals prone to ACL injury, allowing prevention and intervention measures to be applied.

## Background

The rate of noncontact anterior cruciate ligament (ACL) injury has increased substantially among athletes [1]. An increasing number of scholars have begun to pay close attention to targeted prevention and treatment, and early assessment and identification of ACL injury risk factors are necessary to reduce the incidence of this kind of injury and to avoid long-term damage caused by knee joint instability, resulting in cartilage wear and early knee joint osteoarthritis [2].

With the deepening of the understanding of anatomical factors affecting ACL injury, the study of bone morphometry of the tibia and femur has received extensive attention. Medial tibial compartment depression [3] and posterior tibial plateau tilt [4, 5] are associated with increased ACL load and risk of injury. The asymmetry of the medial and lateral compartments with medial tibial concavity and lateral convexity, as well as asymmetry of the distal femoral condylar shape, contribute to the pivoting mechanism [6, 7]. Thus, axial movement occurs, further causing ACL injury [8].

The bony morphology of the lateral femoral compartment is currently of considerable interest because it may play an important role in the phenomenon of pivot shift [9–11]. Studies have suggested that LFCR measured by X-ray plays a role in predicting noncontact ACL injury of the knee joint [12]. Whether LFCR measured by magnetic resonance imaging (MRI) is a risk factor for noncontact ACL injury is unclear. The main purpose of this study was to evaluate the shape of the lateral femoral compartment by MRI and investigate its impact on the risk of noncontact ACL injury. The secondary objective was to evaluate the most predictive risk factor for noncontact ACL injury by comparing LFCR with existing bone morphological risk factors. Our hypothesis was that LFCR, as measured by MRI, was associated with higher risk of noncontact ACL injury.

## Materials and methods

### Study design

**Fig. 1:**
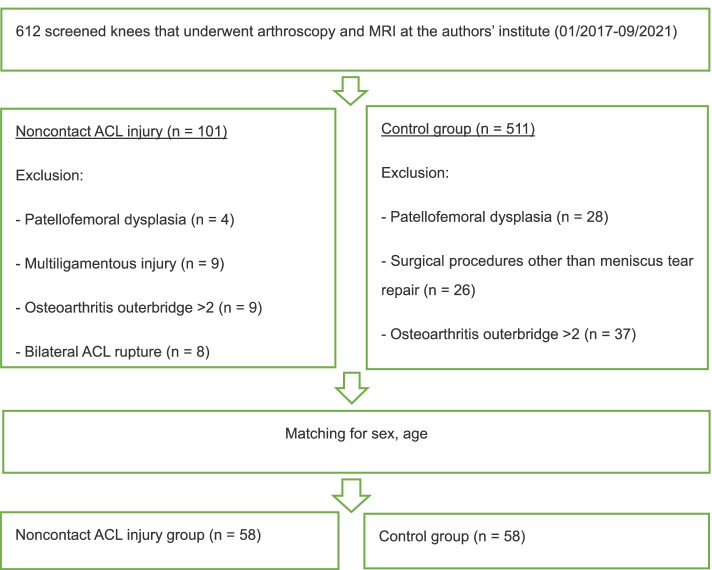
Flowchart and eligibility. ACL, anterior cruciate ligament; MRI, magnetic resonance imaging

A total of 612 patients who underwent arthroscopic surgery for ACL injury or simple meniscus tears or who underwent MRI for anterior knee pain at The Affiliated Central Hospital of Chongqing University from 2017 to 2021 were retrospectively analysed. Patients were divided into the following groups: (1) the noncontact ACL injury group and (2) the control group, which included those with simple meniscus tears without ligament injury and no signs of patellofemoral dysplasia. Patients in the control group were matched by sex and age with those in the treatment group and then assessed with respect to the eligibility criteria listed in Fig. 1. All patients underwent MRI, and scans were read by senior radiologists and surgeons to determine the presence of a ruptured ACL or torn meniscus.

### Instruments and equipment

A 1.5 T MRI scanner (1.5 T, GE Signa, GE Healthcare, USA) was used to measure the knee joint parameters of the study subjects at Central Hospital affiliated with Chongqing University. Sagittal, coronal, and axial sequences of MRI T1 and T2 stages were included, each with a thickness of 3.0 mm.

### MRI data measurement method

**Fig. 2:**
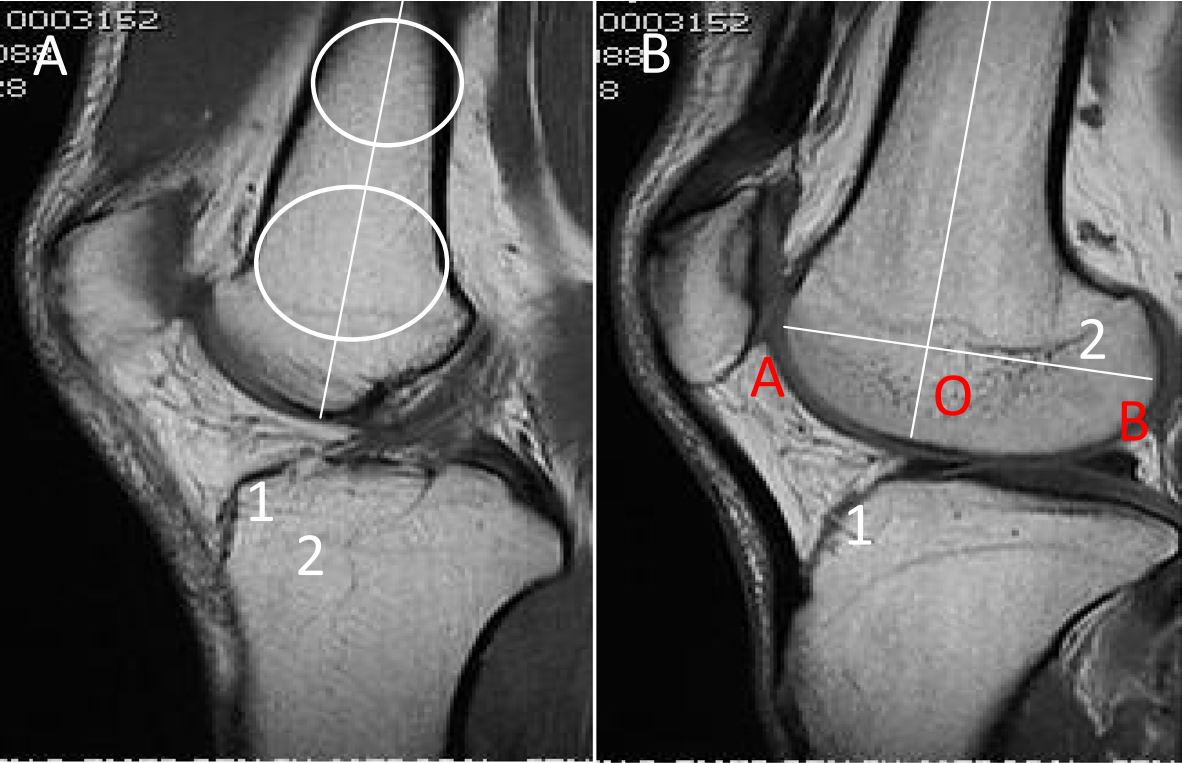
Measurement of LFCR: **A** In the sagittal T1 centre of the knee MRI, 2 circles were drawn in the centre of the femoral axis to determine the long axis of the distal femur. The more distant circle was placed at the nearest end of the tackle. The line passing through the centre of the two circles was considered to be the long axis of the distal shaft of the femur (segment 1). **B** In the sagittal T1 MRI centre of the lateral condyle of the femur, the long axis of the distal femur (segment 1) was replicated. The axis of the femoral condyle was then determined by drawing a line (line segment 2) between the last point of the lateral condyle (point B) and the most anterior point (point A). The distance from the intersection of the two lines (point O) to the last point of the condyle was divided by the total length of the condyle, i.e., OB/AB. This ratio was defined as the lateral femoral condyle ratio

Referring to the method of Pfeifer for X-ray measurement of LFCR [6], LFCR was measured by MRI. First, the sagittal T1 centre of a knee on MRI is selected, in which the insertion of the posterior cruciate ligament, intercondylar spine, and anterior and posterior cortices are convex. To determine the long axis of the distal femur, two circles were drawn at the centre of the femur axis. The more distant circle was placed at the nearest end of the tackle. A line passing through the centre of the two circles was considered to be the long axis of the distal femoral axis. Second, the MRI T1 sagittal centre of the lateral femoral condyle was then selected to replicate the long axis of the distal femur, and the axis of the femoral condyle was determined by drawing a line between the last point and the most anterior point of the lateral condyle. The distance from the intersection of these two lines to the last point of the condyle was divided by the total length of the condyle and multiplied by 100%. This ratio was defined as the lateral femoral condyle ratio (Fig. 2).

**Fig. 3:**
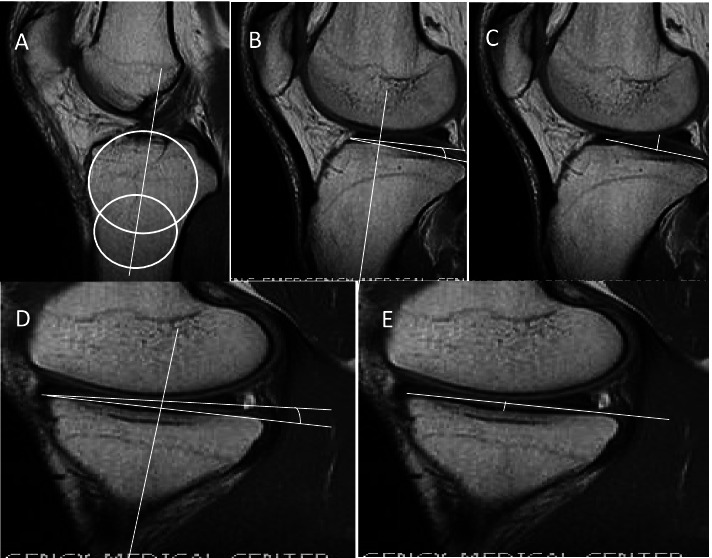
As described previously, various indicators of bone morphology were measured by MRI: **A** midsagittal tibial reference, **B** lateral tibial slope (LTS) [13], **C** lateral tibial height (LTH) [14], **D** medial tibial slope (MTS) [13], and **E** medial tibial depth (MTD) [3]

In this study, bone morphological indicators including the medial tibial slope (MTS) [13], lateral tibial slope (LTS) [13], medial tibial depth (MTD) [3], and lateral tibial height (LTH) [14] were measured by MRI using the methods of previous studies (Fig. 3).

All measurements were made by 2 blind reviewers (HeM, LiJ) to ensure interobserver reliability for the entire cohort. To assess intraobserver reliability, all measurements were repeated 3 weeks later with a reader (HeM).

### Statistical analysis

IBM SPSS 20.0 (IBM Corp. New York, USA) and MedCalc 12.7 (MedCalc Software bvba, Ostend, Belgium) were used for the statistical analyses. The significance level was set at 0.05. Quantitative data are presented as the mean ± standard deviation. The receiver operating characteristic (ROC) curve sample size estimation yielded the required sample size of 56 patients (alpha, 0.05; power, 0.8). The Kolmogorov–Smirnov test was used to assess whether the variables followed a normal distribution. The Wilcoxon rank sum test was used to compare the morphological parameters (LFCR, LTS, LTH, MTD) in the noncontact ACL rupture group and the control group, and the paired T test was used to compare the morphological parameters (MTS) with a normal distribution. There were statistically significant differences in LFCR, LTS, LTH, MTS and MTD. All indicators were included in the single-factor analysis, and indicators with a *P* value < 0.1 were included in binary logistic regression analysis. LFCR and LTH were screened as independent risk factors for noncontact ACL injury, and the prediction probability of the combined LFCR and LTH was calculated. The critical value and area under the curve (AUC) were calculated by ROC curve analysis to evaluate the clinical diagnostic efficacy of the three risk factors (LFCR, LTH, and LFCR+LTH). The AUC values for each risk factor were compared with the Z-test using MedCalc 12.7 software.

### Ethical approval

Informed consent was obtained from all participants or, if participants were under 16, from a parent and/or legal guardian, and the study was approved by the Committee of Central Hospital affiliated with Chongqing University.

## Results

A total of 116 patients were included in the final analysis (58 in the noncontact ACL injury group and 58 in the control group). Each group consisted of 20 females and 38 males, with an average age of 31.05 ± 9.28 years in the noncontact ACL injury group and 30.76 ± 9.38 years in the control group. The intraobserver reliability for LFCR was 0.86 (95% CI, 0.80-0.90), and the interobserver reliability for LFCR was 0.85 (95% CI, 0.79-0.90). The intraobserver reliability for LTS was 0.83 (95% CI, 0.77-0.88), and the interobserver reliability for LTS was 0.85 (95% CI, 0.78-0.90). The intraobserver reliability for LTH was 0.87 (95% CI, 0.82-0.91), and the interobserver reliability for LTH was 0.84 (95% CI, 0.77-0.89). The intraobserver reliability for MTS was 0.89 (95% CI, 0.84-0.92), and the interobserver reliability for MTS was 0.90 (95% CI, 0.85-0.93). The intraobserver reliability for MTD was 0.84 (95% CI, 0.78-0.89), and the interobserver reliability for MTD was 0.82 (95% CI, 0.72-0.88).

LFCR in the noncontact ACL injury group (0.64 ± 0.03) was significantly higher than that in the control group (0.60 ± 0.02) (*P* < 0.01). The LTS, LTH, MTS and MTD in the noncontact ACL injury group were higher than those in the control group (Wilcoxon rank sum tests were used for comparisons of LFCR, LTS, LTH and MTD, and paired T tests were used for comparison of MTS) (Table 1).

**Table 1:** Bone morphological indexes among groups^a^

	Noncontact ACL injury (*n* = 58)	control group (*n* = 58)	*P* value
LFCR^b^	0.64 ± 0.03	0.60 ± 0.02	< 0.01
LTS^b^ (°)	8.13 ± 3.85	6.40 ± 3.77	0.03
LTH^b^ (mm)	2.81 ± 0.76	2.33 ± 0.58	< 0.01
MTS^c^ (°)	7.36 ± 3.45	6.01 ± 3.01	0.046
MTD^b^ (mm)	2.83 ± 0.71	2.59 ± 0.60	0.04

^a^Values are expressed as the mean ± standard deviation. *LFCR* lateral femoral condyle index ratio, *LTS* lateral tibial slope, *LTH* lateral tibial height, *MTS* medial tibial slope, *MTD* medial tibial depth

^b^*P* values refer to the Wilcoxon rank sum test

^c^*P* values refer to the paired T test

Sex differences in LFCR between the noncontact ACL injury group and the control group were not statistically significant. The mean LFCR was 0.64 ± 0.03 in males and 0.63 ± 0.03 in females in the noncontact ACL injury group. In the control group, the mean LFCR was 0.60 ± 0.02 in males and 0.60 ± 0.03 in females.

Single-factor logistic regression analysis was performed to screen potential risk factors, and LFCR (*P* < 0.01), LTS (*P* = 0.02), LTH (*P* < 0.01), MTS (*P* = 0.03), and MTD (*P* = 0.05) were included in the logistic regression equation. Independent risk factors associated with an increased risk of noncontact ACL injury were identified as LFCR (OR, 1.68; 95% CI, 1.35-2.10) (*P* < 0.01) and LTH (OR, 2.65; 95% CI, 1.28-5.46) (*P* < 0.01) (Table 2).

**Table 2:** Univariate and multivariate analyses of all MRI measurements^a^

	Univariate analysis *P* value	Multivariate analysis		
		*P* value	OR	OR:95% CI
LFCR (%)	< 0.01	< 0.01	1.68	1.35-2.10
LTS	0.02	0.49	1.05	0.91-1.22
LTH	< 0.01	< 0.01	2.65	1.28-5.46
MTS	0.03	0.12	1.16	0.96-1.39
MTD	0.05	0.66	1.19	0.55-2.61

^a^*MRI* magnetic resonance imaging, *LFCR* lateral femoral condyle index ratio, *LTS* lateral tibial slope, *LTH* lateral tibial height, *MTS* medial tibial slope, *MTD* medial tibial depth, *OR* odds ratio

**Fig. 4:**
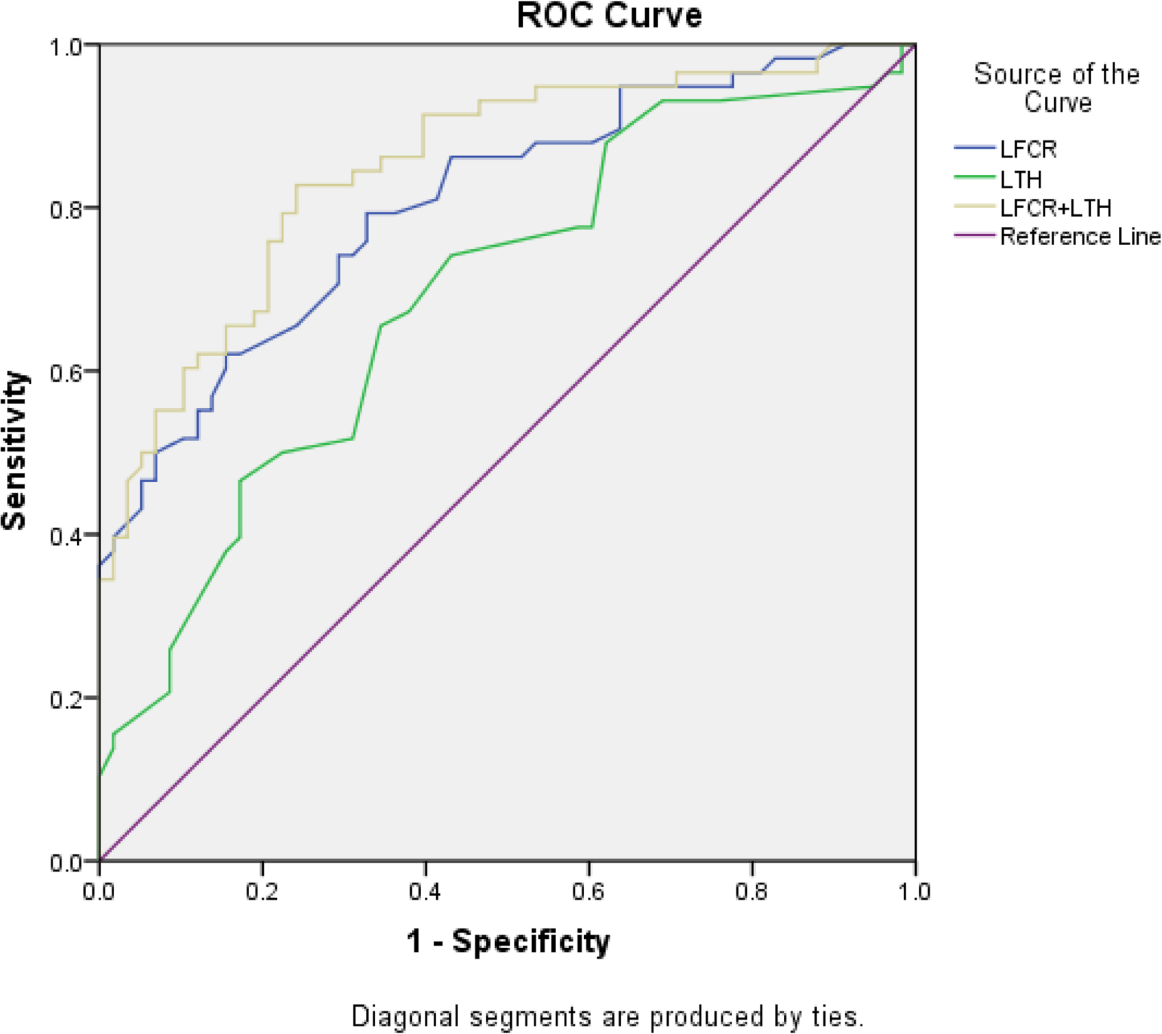
Receiver operating characteristic curves for LFCR, LTH, and LFCR+LTH. Reference line: AUC = 0.5. AUC, area under the curve; LFCI, lateral femoral condyle ratio; LTH, lateral tibial height

LFCR and LTH were screened as independent risk factors for noncontact ACL injury, and the prediction probability of the combined LFCR and LTH was calculated according to logistic regression analysis. ROC analysis showed that all the risk factors exhibited significant accuracy in identifying noncontact ACL injuries (LFCR, LTH, and LFCR+LTH, *P* < 0.01). The AUC for LFCR was 0.81 (95% CI, 0.73-0.88), and the sensitivity and specificity were 0.79 and 0.67, respectively. The maximum of the Youden index (0.47) was calculated to obtain the cut-off value (0.61) of LFCR in the diagnosis process. The AUC for LTH was 0.69 (95% CI, 0.60-0.79), and the sensitivity, specificity and cut-off values for predicting noncontact ACL injury were 0.74, 0.57, and 2.35 mm, respectively (Youden index = 0.31). Of all the risk factors, LFCR+LTH had the highest AUC (0.85; 95% CI, 0.78-0.92), the best specificity (0.83) for noncontact ACL injury, and the highest sensitivity (0.76) for noncontact ACL injury. However, the AUC for LFCR+LTH was not significantly different from that of LFCR alone (*P* = 0.11) (Table 3 and Fig. 4).

**Table 3:** Diagnostic Performance Among the Three MRI Measurements^a^

	Cut-off value	Sensitivity	Specificity	Youden index	AUC	AUC:95%CI	*P* value^b^	*P* value^c^
LFCR	0.61	0.79	0.67	0.47	0.81	0.73-0.88	< 0.01	–
LTH	2.35	0.74	0.57	0.31	0.69	0.60-0.79	< 0.01	0.04
LFCR+LTH	0.46	0.83	0.76	0.59	0.85	0.78-0.92	< 0.01	0.11

^a^*MRI* magnetic resonance imaging, *LFCR* lateral femoral condyle ratio, *LTH* lateral tibial height, *AUC* area under the curve

^b^*P* value of each AUC tested against 0.5; binomial Z-test

^c^*P* value of each AUC tested against the AUC for the LFCR; binomial Z-test

## Discussion

The current study found that an increased LFCR, as measured by MRI, was associated with an increased risk of noncontact ACL injury. The LFCR threshold for predicting noncontact ACL rupture was 0.61, with a sensitivity of 0.79 and specificity of 0.67, which confirmed the hypothesis of the study. Combining the two most predictive factors (LFCR> 0.61 and LTH > 2.35 mm) improved the predictive diagnostic performance for noncontact ACL injury. The AUC was 0.85, sensitivity was 0.83, and specificity was 0.76. These parameters can help clinicians identify patients at risk for ACL injury.

Many studies have shown that the shape of the lateral femoral condyle exerts an important influence on the rotational stability of the knee [9, 14]. Pfeiffer reported that the mechanism of the influence of lateral femoral condyle shape on knee rotation stability was an increase in the depth of the lateral femoral posterior condyle, which may affect the movement of the femur in relation to the tibia and lead to changes in gait and load mechanics [8]. The increase in the depth of the lateral posterior condyle of the femur alters the shape of the lateral condyle of the femur to be more elliptical and not equidistant, which may lead to an increase in ligament relaxation when the knee joint is nearly fully extended [8]. It may also lead to a reduction in the contact area between the femur and tibia, thereby increasing rotational relaxation of the knee [8]. The results of this study are consistent with the aforementioned results.

A steeper tibial slope was significantly correlated with an increase in axial displacement [15–17] and with the risk of ACL injury [15–17]. Interestingly, in this study, only a significant increase in tibial slope was found in the noncontact ACL injury group compared to the control group. However, there have also been literature reports that found no association between steeper tibial slopes and a higher risk of ACL injury [18, 19].

In this study, we found that the combination of the two factors (LFCR> 0.61, LTH > 2.35 mm) was more predictive for noncontact ACL injuries than either factor alone. This confirms previous findings that bone morphology of both the femoral and tibial lateral ventricles contributes to axial displacement of the knee. That is, increased depth of the lateral posterior condyle of the femur and height of the lateral tibial plateau lead to increased rotation [9, 20]. These differences in bone morphology affect the biomechanics of the knee, causing greater traction of the ACL and increasing the risk of injury [21].

In this study, there was no significant difference in LFCR between women and men. In previous studies, sex differences in the morphology of the distal femur were reported. Some studies found that the anatomical morphology of femurs in females was inconsistent with that of femurs in males [11, 22], while other studies found that there was no significant difference in the morphology of distal femurs between males and females [10].

Previous studies have used different measurement methods to describe the bony morphology of the lateral femoral condyle and further found that bone morphology changes in the lateral femoral condyle could increase the probability of ACL injury. Pfeiffer quantitatively measured the osseous morphology of the lateral condyle of the femur according to the ratio of the lateral condyle on X-ray [11]. This method was simple and relatively accurate. The exclusion of a large number of patients because of malrotated radiographs could introduce bias. The study by Voleti et al. also demonstrated that radiographs underestimate posterior condylar depth measurements when compared with MRI [23]. The use of MRI would have allowed the authors to accurately quantify the posterior condylar depths of the lateral femoral condyles, assess their influence on the risk of ACL injury and reduce measurement imprecision and patient exclusion because of malrotated radiographs. Hodel et al. used the LFCI to quantitatively measure the skeletal morphology of the lateral femoral condyle on MRI [10]. In other words, the flexion circle and extension circle were drawn before and after the lateral femoral condyle, respectively, and the ratio of the radius of the two circles was considered the lateral femoral condyle index [10]. However, Li et al. indicated that the two circles had great uncertainty and randomness, possibly leading to large errors of this measurement method [24].

This study offers some strengths but also presents limitations. The main advantage of this study is that the LFCR was measured by MRI, and this method is simple to apply. Reducing the exclusion of large numbers of patients due to poor X-ray rotation may introduce bias. Considering several risk factors, a diagnostic threshold was proposed. The primary limitation of this study is that only conventional MRI was used to measure the morphological parameters of this study, instead of 3D-MRI examination. When morphological parameters are measured, the ideal sagittal section should be perpendicular to the line connecting the posterior condyle of the femur [25], which is difficult to ensure with conventional MRI [26]. Conventional MRI with excessive section thickness will make it difficult to accurately identify the sections and points of interest, resulting in errors [27]. Compared with conventional MRI, 3D-MRI can perform high-resolution isotropic acquisition through unspaced thin layer scanning [28] and create multi-plane recombination images on any angle plane while reducing some volume artefacts, thus solving the problems of image presentation. The second limitation of this study is that the control group included a group of people who visited the hospital for anterior knee pain, not healthy people. Therefore, they could have higher risk of knee sprain with respect to the general population, potentially leading to underestimation of the investigated parameters.

## Conclusion

In this study, an elevated LFCR was associated with noncontact ACL injury as detected by MRI, and the LFCR and LTH were the most predictive risk factors for noncontact ACL injury. This may help clinicians identify individuals prone to noncontact ACL injury, allowing targeted prevention measures and interventions.

## Data Availability

All raw data and materials utilized during the study are available from the first author by request (He Miao, smallhem@163.com).

## Abbreviations

LFCR: Lateral femoral condyle ratio
ACL: Anterior cruciate ligament
MRI: Magnetic resonance imaging
LTS: Lateral tibial slope
LTH: Lateral tibial height
MTS: Medial tibial slope
MTD: Medial tibial depth
AUCs: Areas under the curve
ROC: Receiver operating characteristic
